# FDG-PET in Follicular Lymphoma Management

**DOI:** 10.1155/2012/370272

**Published:** 2012-07-30

**Authors:** C. Bodet-Milin, T. Eugène, T. Gastinne, E. Frampas, S. Le Gouill, F. Kraeber-Bodéré

**Affiliations:** ^1^Nuclear Medicine Department, University Hospital-ICO-Gauducheau, INSERM UMR 892 Team 13, 44093 Nantes, France; ^2^Hematology Department, University Hospital, INSERM UMR 892 Team 10, 44093 Nantes, France; ^3^Radiology Department, University Hospital, INSERM UMR 892 Team 13, 44093 Nantes, France

## Abstract

18-Fluoro-deoxyglucose positron emission tomography/computerised tomography (FDG PET/CT) is commonly used in the management of patients with lymphomas and is recommended for both initial staging and response assessment after treatment in patients with diffuse large B-cell lymphoma and Hodgkin lymphoma. Despite the FDG avidity of follicular lymphoma (FL), FDG PET/CT is not yet applied in standard clinical practice for patients with FL. However, FDG PET/CT is more accurate than conventional imaging for initial staging, often prompting significant management change, and allows noninvasive characterization to guide assessment of high-grade transformation. For restaging, FDG PET/CT assists in distinguishing between scar tissue and viable tumors in residual masses and a positive PET after induction treatment would seem to predict a shorter progression-free survival.

## 1. Introduction

Follicular lymphoma (FL) is one of the most common types of lymphoma, representing around 25% of adult non-Hodgkin lymphomas (NHLs) worldwide [1]. FL evolution is highly variable with differences in clinical presentation, histological appearance, clinical behaviour, and response to therapy. Indeed, while some FL patients achieve prolonged complete remission (CR) other experience iterative relapses with or without histological transformation into a high-grade lymphoma (25–60%) [2, 3]. Treatment options, including watchful waiting, external radiotherapy, chemotherapy, monoclonal antibodies, radioimmunotherapy (RAIT), and biologic therapies, are guided by clinical features, the extent of disease at presentation and prognostic indices such as the FL international prognostic index (FLIPI) [4–6]. In order to segregate between patients with an indolent FL from those with more aggressive disease, risk stratification and identifying factors predictive of survival are of major interest in this disease.

18-Fluoro-deoxyglucose positron emission tomography (FDG PET/CT) is a noninvasive whole-body tri-dimensional imaging technique. FDG PET/CT is commonly used in the management of patients with lymphomas especially for initial staging and response assessment at the end of treatment in patients with diffuse large B-cell lymphoma (DLBCL) and Hodgkin lymphoma (HL). Despite the now well recognized FDG avidity of FL, the use of FDG PET/CT is not recommended in standard practice [7–9]. Herein, we review using recent publications, the interest of FDG PET/CT in FL and the potential of new PET tracers such as radio-labeled monoclonal antibodies (MAbs).

## 2. FDG PET for Initial Staging

In order to stage the disease (Ann Arbor classification) and calculate the FLIPI score, initial evaluation for FL includes physical examination, haematological and biochemical analysis, CT imaging of chest, abdomen and pelvis, plus bone marrow biopsy [10]. Outside clinical trial where FDG PET/CT is recommended before therapy principally to improve posttherapy evaluation, FDG PET/CT at time of diagnosis is not routinely performed [7]. Nevertheless, FDG PET/CT at diagnosis may be of interest not only for disease staging or posttherapy evaluation but also to guide the therapeutic strategy in FL.

A primary example of the utility of FDG PET/CT would be with an FL patient presenting at diagnosis with conventionally staged localized disease. FDG PET/CT could be of great interest to confirm that there is a unique site involved. Then, the use of radiation instead of a wait and watch option may be a reasonable therapeutic approach. Many retrospective studies have shown that FDG PET/CT detects more lesions than CT scans, especially lymph node involvement and extranodal lesions. The ability of FDG-PET to evaluate bone marrow infiltration in patients with lymphoma has also been investigated extensively. Prior studies showed that FDG-PET has a high potential to detect bone marrow involvement in high-grade malignant lymphoma but had low sensitivity for the detection of diffuse bone marrow infiltration in low-grade NHL especially FL [11]. Despite this, in retrospective study, initial FDG PET examination modified Ann Arbor staging in 11 to 31% of patients, particularly for patients considered as early stage on standard evaluation [12–19]. In a series of 45 untreated FL patients, Le Dortz et al. reported that 11% of patients considered early stage (I/II) following standard evaluation (physical examination, CT, and bone marrow biopsy) were found to be having advanced III/IV stage when FDG PET/CT was taken into account [20]. This aspect appears influential in choosing an optimal initial therapeutic strategy.

**Figure 1:**
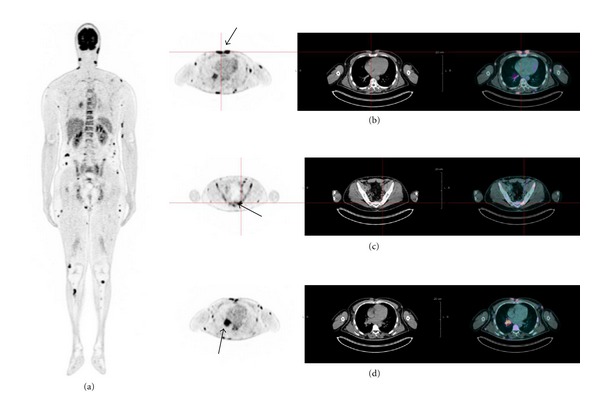
The FDG PET/CT images of a patient with rapidly progressive cutaneous follicular lymphoma presenting general signs (a). CT scans showed cutaneous nodes and hilar lymph nodes. Bone marrow biopsy was negative. FDG PET/CT was realised in order to detect systemic lymphoma and to guide biopsy to detect aggressive transformation. FDG PET/CT shows high levels of fixation in subcutaneous nodes, especially in presternal area (with maximal SUV of 25) (b), in supra-and infradiaphragmatic lymph nodes (c) and in focalized bone foci (d). Biopsy realised in presternal area confirmed aggressive transformation of FL.

A second major value of FDG PET/CT at diagnosis is to guide diagnostic biopsy in the most FDG-avid site of disease in FL patients showing clinical, biochemical, or anatomical signs of aggressive transformation. Histological transformation of indolent lymphoma is a dramatic event that occurs in 5–10% of patients and carries a poor prognosis [21]. Identification of patients with histological transformation often leads to a change in therapeutic management requiring intensified immuno-chemotherapy regimens. Moreover, patients with histological transformation achieving a complete response after intensified chemotherapy can experience prolonged survival, indicating the benefits of detecting histological transformation as early as possible [22]. Schöder et al. were the first to show, in a series of 97 patients, that despite an overlap between indolent and aggressive disease in the low SUV range, all cases of indolent lymphoma had an SUVmax ≤13. They found a sensitivity and specificity to detect aggressive lymphoma of 71% and 81% using a SUV cut-off of 10 [23]. In a prospective study including 38 patients with low grade lymphoma, including 23 FL, our group showed that a SUVmax < than 11.7 was always associated with indolent lymphoma, whereas a SUVmax >17 was always associated with transformation [24]. In our series, the SUVmax threshold of 14 was the best compromise regarding correctly classified frequency.  Figure 1 shows an example of a patient included in this study. It was a patient with rapidly progressive cutaneous follicular lymphoma with B symptoms. CT scan showed cutaneous nodes and hilar lymph nodes. The bone marrow biopsy was negative. FDG PET/CT was performed in order to detect systemic lymphoma and to guide biopsy to identify aggressive transformation. FDG PET/CT showed marked FDG uptake in subcutaneous nodes, especially in the presternal area (with maximal SUV of 25) in supra and infradiaphragmatic lymph nodes and in focalized bone foci. Biopsy performed in the presternal area confirmed an aggressive transformation of FL. In this patient, FDG PET/CT guided biopsy in the most FDG-avid site of disease and modified the extent of the disease indicating pathologic bone foci whereas the bone marrow biopsy was negative. This study concurs with others reports suggesting the prognostic value and therapeutic impact of FDG PET/CT to guide biopsy in the most FDG-avid site of disease in FL patients with either very high or low SUVmax. However the SUV cut-off values distinguishing between indolent or aggressive lymphoma were different in all studies [24–26]. These differences in SUV cut-off values highlighting the limitation of semi-quantitative analysis using SUVmax. SUVmax measurement could be influenced by many factors including injected dose, time between injection and imaging, patient weight, blood glucose concentration, time per step, partial volume effects and reconstruction parameters. News parameters like SUVpeak or metabolic tumor volume (MTV) and total glycolytic lesion index (TLG) should be assessed but may be more reproducible, robust, and accurate in lymphoma evaluation [27, 28].

## 3. FDG PET for Response Assessment at the End of Therapy

According to the IHP, response assessment using FDG PET/CT is not routinely recommended for FL assessment at the end of therapy. FDG PET/CT may be useful for more accurate determination of response in clinical trials, if FDG PET/CT is positive prior to treatment and when overall response and particularly complete response (CR) rates are major end-points of the clinical study [7].

It is well known that response assessment to treatment using anatomic imaging has limitations. Persistence of residual masses after chemotherapy does not necessarily indicate residual disease and anatomic imaging has limited ability to distinguish between scar tissue and viable tumors in residual masses. Characterization of such residual masses is problematic in patients with FL because a significant proportion of patients with FL will present residual masses (partial responder (PR) or unconfirmed complete responder (CR) according to 1999 International Workshop Criteria IWC) after treatment. Of these, ≤20% will present true residual disease.

In HD or DLBCL, it has been demonstrated that a positive FDG PET/CT after treatment completion is a poor prognostic factor [29–31]. The question of prognosis has been unanswered in FL but recent findings have provided clarity. In a retrospective study performed in a series of 91 FL patients, Lopci et al. showed that FDG PET/CT is an accurate imaging modality for the assessment of treatment response in FL, with high rates of sensitivity (100%), specificity (99%), positive predictive value (89%), and negative predictive value (100%) [32]. The study population was heterogeneous, especially with respect to the number of prior therapies, and FDG PET/CT scans were not analyzed using IHP criteria. However, univariate Kaplan-Meier analysis demonstrated a statistically significant correlation with progression-free-survival (PFS) for postinduction FDG PET (*P* < 0.0001), FLIPI score (0-1 versus ≥2) (*P* = 0.0451) and number of relapses (none versus ≥1) (*P* = 0.0058). On multivariate analysis only FDG PET/CT (*P* = 0.0006892) and number of relapses (*P* = 0.01947) were independent predictive factors for PFS. Another retrospective study performed by Le Dortz et al. on 45 untreated biopsy-proven FL patients reported good performance of FDG PET/CT analyzed according to IHP criteria, defining 100%-sensitivity and 97%-specificity for residual disease detection after induction treatment with rituximab combined with CHOP [20]. PFS for patients with negative post-treatment PET was 48 months (95% CI: 42.6–53.5), as compared to 17.2 months (95% CI: 9.4–25) for those with positive post-treatment PET (*P* < 0.0001).

More recently, Trotman et al. published the prognostic significance of FDG PET/CT performed after first-line therapy in a larger series of FL patients treated in the prospective PRIMA study [33]. Results of 122 FDG PET/CT performed after induction immunochemotherapy were recorded retrospectively and patients went on to either observation or rituximab maintenance per protocol independent of the FDG PET/CT result. In this study, positive or negative FDG PET was defined by the local investigator's interpretation of the nuclear medicine physician's scan report without independent scan review. According to these criteria, 26% of scans were considered as positive at the end of the induction and metabolic response was correlated with conventional response criteria (*P* < 0.001). Initial demographics or disease characteristics did not differ between FDG PET-positive and PET-negative patients. Patients remaining PET positive had a significantly (*P* < 0.001) lower PFS at 42 months of 32.9% (95% CI, 17.2% to 49.5%) compared to 70.7% (95% CI, 59.3% to 79.4%) in patients with PET negative. FDG PET/CT status, but not conventional response (CR or unconfirmed CR versus partial response (PR)) according to 1999 International Workshop Criteria (IWC), was an independent predictive factor for progression. Moreover, the risk of death was also increased in FDG PET-positive patients (hazard ratio 7.0; *P* = 0.0011). Similar results were obtained when the different FLIPI categories were included, but FLIPI status itself was not significantly predictive of outcome alone in this small cohort.

**Figure 2:**
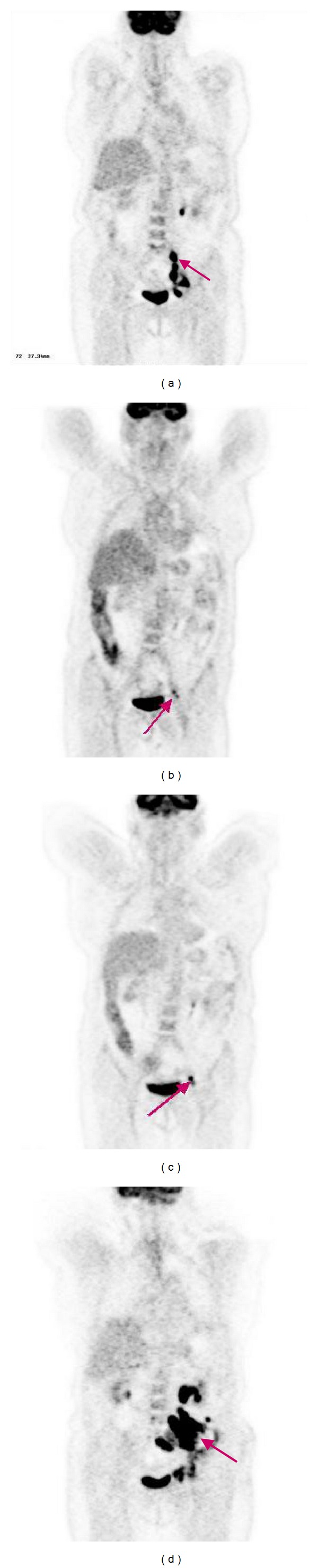
FDG PET/CT imaging of a patient with a stage 2 FL in relapse, treated by RAIT in a phase I/II protocol using fractioned ^90^Y-epratuzumab with a partial metabolic response at 6 weeks and 3 months after RIT and who experienced relapse 6 months after RAIT.  Figure 1(a) shows the FDG PET/CT image before RAIT with accumulation of FDG in lomboaortic, iliac, and inguinal nodes. Figures 1(b) and 1(c) show the FDG PET/CT images realised 6 weeks and 3 months after RIT, showing a residual uptake of FDG in left iliac nodes.  Figure 1(d) shows the FDG-PET images realised 6 months after RIT with pathologic accumulation of FDG in lombo-aortic, iliac and inguinal nodes confirming clinical suspicion of relapse.

FDG PET/CT has also shown to be of utility in evaluation of response following radioimmunotherapy (RAIT). In this indication, if an early assessment of disease seems to have a prognosis impact, the optimal time to perform FDG PET/CT studies after RIT is always discussed and need to be determined. Lopci et al. studied the role of FDG PET/CT evaluation 3 months after ^90^Y-ibritumomab tiuxetan (Zevalin) in 59 patients with relapsed or refractory FL patients [34]. In this series, FDG PET concluded to 46% of CR, 25% of PR and 29% of nonresponders with an overall survival of 71.2%. With a median follow-up period of 23 months, the univariate analysis showed a statistically significant relation between FDG PET/CT response to RAIT and PFS (*P* = 0.015), while all the other prognostic factors showed no significant correlation. FDG PET/CT was the only independent predictor of PFS on multivariate analysis (*P* < 0.001). Our group demonstrated that a positive assessment of disease by FDG PET/CT performed as soon as six weeks after therapy corresponded with a shorter time to progression in patients receiving fractionated RAIT using anti-CD22 ^90^Y-epratuzumab [35]. In this series of 27 patients, including 16 FL, the mean time to progression was 15.6 months when FDG PET/CT was negative for disease, compared to 5.4 months when FDG PET/CT was positive (*P* = 0.008).  Figure 2 shows FDG PET/CT images in an FL patient included in this study, with a FDG PET/CT-positive result after treatment (unconfirmed CR according to IWC CT morphological evaluation), and shows a short duration response after the end of the treatment.

Taken together, all these studies provide promising preliminary data. However, further standardized research is needed because most of the previous published series are retrospective, performed on a heterogeneous population, with different therapeutic regimens and without standardized FDG PET/CT acquisition and reporting criteria. In addition, the relapse rate in FL patients is higher than that of other more “aggressive” lymphoma and longtime followup will be required to determine if FDG PET/CT response is predictive of overall survival rather than just time to progression. The long natural history of FL and the potential efficacy of the second and other lines of treatment mean that differences in PFS do not always translate into differences in survival. Moreover, what is the potential therapeutic impact of FDG PET response? Current front line therapy for advanced FL is not curative. FDG PET/CT may distinguish between patients destined to have longer or shorter remission, but cannot distinguish a cured from a noncured population. Specific standardized criteria should probably be proposed in FL, on one hand to identify after induction therapy those patients with high-risk of short response duration, requiring an intensive therapy and on the other to avoid false positive results so as to improve the specificity and positive predictive value of FDG PET/CT.

## 4. FDG PET for Midtreatment Response**** Evaluation

In aggressive lymphoma patients under chemotherapy, early FDG PET/CT response evaluation assists in distinguishing rapid and slow responders, providing the opportunity for escalate in slow-responders and potentially improve outcomes or deescalation in rapid responders and potentially decrease adverse events of treatment. According to the IHP publications, no indication of early metabolic assessment has yet been validated in clinical practice. In DLBCL and HL patients, different studies have shown the prognostic value of early FDG PET/CT response, after 1 to 4 courses of chemotherapy, using visual criteria or variation of SUVmax and several clinical trials remain on going, assessing interim-PET-based-therapy after 2 or 4 courses, in particular in advanced DLBCL and HL disease [36–45]. In FL patients, the data is very limited and the clinical relevance appears less clear because treatment intent is rarely curative as previously explained. Bishu et al. assessed the role of a midinduction FDG PET in 31 patients with grade I-II FL [19]. Patients with positive FDG PET at the end of induction had shorter mean PFS of 5.8 months compared to 29.5 months for PET-negative patients (*P* < 0.01). In contrast, midtherapy FDG PET was not significantly correlated with outcome. These results suggested that a midtherapy escalated decision did not appear relevant whereas de-escalation or a decrease in the number of chemotherapy courses could be proposed if a rapid normalization of FDG PET is observed. This question should probably be relevant for a randomized clinical study in low risk FL patients. However such study in low risk FL patients is unlikely to be feasible given the numbers of patients required to demonstrate any impact on survival.

## 5. FDG PET and Detection of Relapse

FL is almost invariably associated with slow but continuous relapse. Given the indolent nature of this disease, long-term monitoring is needed but few studies have explained the utility of different follow-up approaches to detect the recurrence of follicular lymphoma, especially in stages I–III. According to the European Society of Medical Oncology (ESMO) working group, recommendations for follow up in FL include physical examination, blood count, routine chemistry, and minimal radiological exam or ultrasound. Regular CT scans to screen residual disease or to detect pre-clinical relapse should not be systematically performed outside of clinical trials [4]. In the IHP, FDG PET is not recommended in the followup of FL as in other subtypes of lymphoma [7]. According to its high sensitivity, FDG PET/CT in followup of FL patients could lead to false positive results potentially responsible for further investigations and complications. FDG PET/CT in the followup would also probably lead to earlier diagnosis of recurrence, sometimes in asymptomatic patients. The impact of an early detection of relapse on overall survival is unclear in this chronic disease and will perhaps need to be addressed in prospective randomized studies, especially in patients with potential therapeutic impact, for example, in patients treated by maintenance Rituximab in first line therapy or first relapse.

## 6. Perspectives with Immuno-PET

A major limitation associated with FDG PET/CT is nonspecific uptake in inflammatory or infectious lesions, and variable physiological uptakes in normal tissues/organs that can be confused with malignancy. One new perspective of approach to overcome these limitations is to use ligands/vectors that can specifically target cell-surface markers, such as receptors or antigens. Phenotypic PET imaging is a promising alternative approach to obtain a specific noninvasive characterization of malignancies by whole-body imaging. In the new context of personalized medicine, several recent developments are contributing to a renewed interest in MAb such as phenotypic imaging agents for immuno-PET. Therefore, the tracking and quantification of MAbs with PET could be an interesting new option to better understand the efficacy of MAbs in individual patients. Successful implementation of immuno-PET requires access to exotic PET radionuclides such as ^124^I, ^64^Cu, or ^89^Zr for labeling of intact immunoglobulin. In lymphoma, MAb targeting CD20 or CD22 are available and could be used for immuno-PET purposes and the feasibility of immuno-PET using anti-CD20 MAb has been demonstrated [45–47]. High sensitivity and specificity PET imaging is awaited using immuno-PET in B lymphoma, as already demonstrated in different types of solid tumor, such as renal, head and neck, or breast carcinoma [48–51].

## 7. Conclusion

While the use of FDG PET/CT imaging remains invalidated in the current management of FL, recent publications have suggested benefits in numerous situations. FDG PET/CT at diagnosis seems to have two advantages in FL, firstly for disease staging with better accuracy than conventional imaging and secondly to guide surgical biopsy in the most FDG-avid site of disease in FL patients showing symptomatic, biochemical or anatomical signs of aggressive transformation. These two aspects appear influential in determining an optimal initial therapeutic strategy. Promising recent studies have suggested that a positive FDG PET/CT after treatment completion of FL conveys a poor prognostic akin to that in HL or DBCL. Further standardized research is needed to confirm the prognosis and therapeutic impact of FDG PET/CT in this lymphoma with natural long history and with high rate of relapse.
